# Architecture of population-differentiated polymorphisms in the human genome

**DOI:** 10.1371/journal.pone.0224089

**Published:** 2019-10-17

**Authors:** Maulana Bachtiar, Yu Jin, Jingbo Wang, Tin Wee Tan, Samuel S. Chong, Kenneth H. K. Ban, Caroline G. L. Lee

**Affiliations:** 1 Department of Biochemistry, Yong Loo Lin School of Medicine, National University of Singapore, Singapore; 2 Division of Cellular & Molecular Research, Humphrey Oei Institute of Cancer Research, National Cancer Centre Singapore, Singapore; 3 National Supercomputing Centre Singapore, Singapore; 4 Department of Pediatrics, Yong Loo Lin School of Medicine, National University of Singapore, Singapore; 5 Cancer & Stem Cell Biology Programme, Duke-NUS Graduate Medical School, Singapore; University of Iceland, ICELAND

## Abstract

Population variation in disease and other phenotype are partly attributed to single nucleotide polymorphisms (SNPs) in the human genome. Due to selection pressure, two individuals from the same ancestral population have more genetic similarity compared to individuals from further geographic regions. Here, we elucidated the genomic population differentiation pattern, by interrogating >22,000,000 SNPs. Majority of population-differentiated (pd) SNPs (~95%), including the potentially functional (pf) (~84%) subset reside in non-genic regions, compared to the proportion of all SNPs (58%) found in non-genic regions. This suggests that differences between populations are more likely due to differences in gene regulation rather than protein function. Actin Cytoskeleton, Axonal Guidance and Protein Kinase A signaling pathways are enriched with genes carrying at least three pdSNPs (enriched pdGenes), while Antigen Presentation, Hepatic Fibrosis and Huntington Disease Signalling pathways are over-represented by enriched pf-pdGenes. An inverse correlation between chromosome size and the proportion of pd-/pf-pdSNPs was observed. Smaller chromosomes have relatively more of such SNPs including genes carrying these SNPs. Genes associated with common diseases and enriched with these pd-/pfpdSNPs are localized to 11 different chromosomes, with immune-related disease pd/pf-pdGenes mainly residing in chromosome 6 while neurological disease pd/pf-pdGenes residing in smaller chromosomes including chromosome 21/22. The associated diseases were reported to show population differences in incidence, severity and/or etiology. In summary, this study highlights the non-sporadic nature of population differentiation footprint in the human genome, which can potentially lead to the identification of genomic regions that play roles in the manifestation of phenotypic differences, including in disease predisposition and drug response.

## Introduction

Each individual is unique, and differs from another individual in many aspects including skin and eye color, disease susceptibility and even immunity. These variations become more obvious between individuals from disparate geographic locations. Two individuals from the same population are more similar compared to those originating from population in a different region or continent. Such phenotypic differences amongst populations can be attributed to the diverse factors encountered during evolution, as reflected by the presence of genomic differences across populations.

Single Nucleotide Polymorphisms (SNPs) are the most abundant form of genetic variation in the human genome, with more than 100 million validated SNPs recorded in the dbSNP database [1]. Due to different natural selection pressures encountered by different populations, SNPs that are associated with phenotypic diversity may exhibit differences in allele frequencies amongst different populations. Different environmental factors in different geographic regions impose different selection forces to either negatively or positively select SNPs that are associated with disadvantageous or advantageous traits, respectively [2]. Hypothetically, these phenomena would leave a ‘genomic footprint’ that can be deciphered by studying the pattern of population differentiation of SNPs across the human genome [2]. For example, negative selection tends to decrease the level of population differentiation [3], while positive selection is associated with increased population differentiation[4].

Depending on the genomic location, SNPs residing within genes can potentially affect gene function and lead to phenotypic differences in different populations. Coding region SNPs, particularly those that are associated with altering the amino acid sequence of a protein, can potentially affect protein function through variation in its structure, activity or post-translational modification activity [5–7]. Moreover, SNPs in regulatory sites including those that affect the transcription factor binding sites on promoters, miRNA binding sites at 3’ un-translated regions (3’UTR), or exon/intron splicing regulatory sites, are implicated in the regulation of gene expression [8, 9].

In this study, we aim to decipher the pattern of population differentiation in the human genome and evaluate its potential implication in affecting gene function, and ultimately phenotype. Using the 1000 Genomes project data [10], population differences in allele frequencies were examined for ~22 million SNPs in 14 global populations representing four different continents: Latin America, Europe, Africa, and East Asia. Population-differentiated (pd) SNPs (pdSNPs) and genes (pdGenes), as well as a subset of these pdSNP/pdGenes that were predicted to be potentially functional (pf-pdSNPs/pf-pdGenes) were identified. To facilitate hypothesis generation and future detailed investigation, pathways as well as diseases enriched with these pd/pf-pdGenes were identified. Literature was interrogated to determine if diseases enriched with pf-pdGenes were also reported to show differences in disease incidences / manifestation. This may then pave the way for the identification of genomic regions that play roles in the manifestation of phenotypic differences, including in disease predisposition and drug response as well as potential target genes/variants that play significant role in phenotype differences.

## Materials and methods

### Estimating population differentiation from genomic data

SNP data from 1,092 individuals originating from 14 populations were downloaded from Phase I of the 1000 Genomes Project [10]. After removal of mono-allelic SNPs that occur in any population, population differentiation of >22,000,000 SNPs were estimated through genome-wide computation of population pairwise *F*_ST_ based on their allele frequency [11, 12]. In each population pair, SNPs with *F*_ST_ score within the top 1% were considered to be population-differentiated and named ‘pdSNPs’ (pdSNP F_*ST*_ scores range from 0.023 to 0.498; median 0.292). A total of 3,168,863 pdSNPs were identified from 91 population pairs.

### Genome-wide SNPs mapping to functional gene regions

Using NCBI Genome Build 37, SNPs were mapped to various genic regions including promoter (5kb upstream transcription start site), 5’UTR, 3’UTR, coding region and intron. SNPs outside the genic regions were labelled as ‘intergenic’ SNPs. Approximately 90,000 pdSNPs were annotated as potentially functional using the pfSNP resource (http://pfs.nus.edu.sg/) [13] and referred to as pf-pdSNPs. The population differentiated genes (pdGenes) were defined as genes that carry at least one pdSNP while pf-pdGenes are genes that carry one or more pf-pdSNPs. Furthermore, genes carrying at least three pdSNPs and pf-pdSNPs were defined as enriched pdGenes and enriched pf-pdGenes, respectively.

To control for linkage disequilibrium (LD) structure, a pruning step was performed for pdSNPS and pf-pdSNPs using PLINK 1.9 [14] ‘—indep-pairwise’ command with the following parameters: window size 1 million bases and r^2^ 0.8. In the pruned dataset, no SNP pair with high LD (r^2^>0.8) was observed.

### Gene-set enrichment analysis

The Ingenuity^®^ Pathway Analysis (IPA^®^) was utilized to identify biological pathways that are over-represented by enriched pdGenes and/or enriched pf-pdGenes. Furthermore, to determine the diseases associated with chromosome-specific over-representation of enriched pdGenes and/or pf-pdGenes, the *enrichDO* function within the *ClusterProfiler* [15] R package were utilized. Disease ontology (DO) annotation of genes was obtained from DOSE R package [16]. With this tool, gene-set enrichment analysis was performed based on Disease Ontology (DO) classification and hypergeometric modeling. The resulting P-values were adjusted using false discovery rate (FDR) multiple test correction. Disease ontology with adjusted p-value<0.05 were identified to be enriched by the specific gene set e.g. enriched pd-/pf-pdGenes. The *compareCluster* function was utilized to compare the results across all the human chromosomes.

## Results

### Distribution of population differentiated SNPs

**Fig 1:**
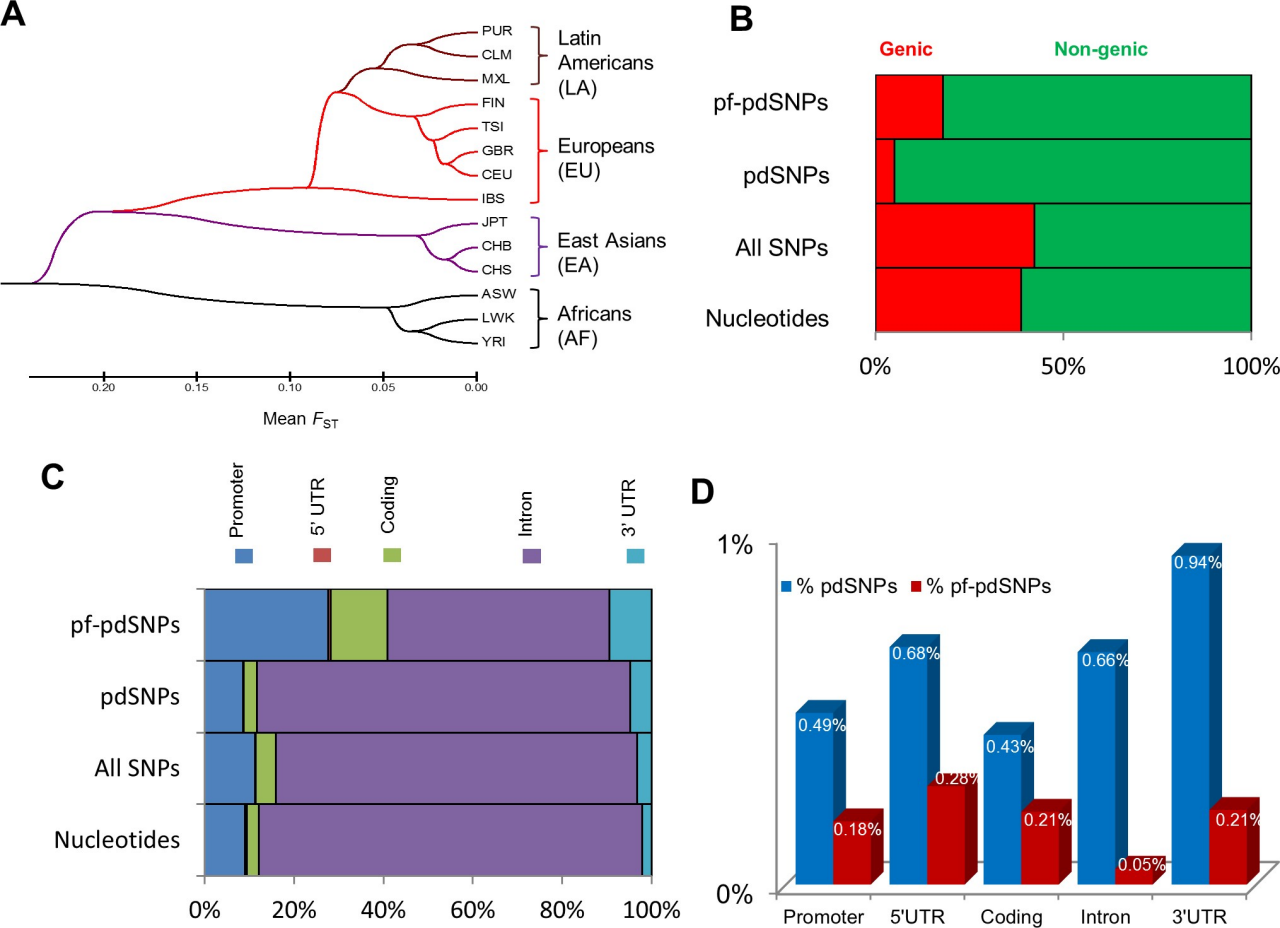
Distribution of population differentiated (pd) and potentially functional population differentiated (pf) pf-pdSNPs in the genome. (A) A population tree constructed using the average pairwise *F*_ST_ scores of the SNPs within the top 1% of the population pair *F*_ST_ distribution, referred as the ‘pdSNPs’. (B) The composition of different SNP categories in the human genome. (C) The proportion of genic SNPs found in the promoter, 5’ UTR, coding, intron, and 3’ UTR regions. (D) The percentage of pdSNPs (blue bar) and pf-pdSNPs (red bar) observed in the different gene regions.

To decipher the pattern of population differentiation, SNPs that are population differentiated (pdSNPs) were identified through computation of SNP *F*_ST_ scores across 91 population pair combinations. The *F*_ST_ population differentiation scores of these pdSNPs appropriately cluster the various populations phylogenetically (Fig 1A). The distribution and genomic nucleotides composition of pdSNPs and pf-pdSNPs were then examined. Approximately 42% of SNPs in the human genome are found in genic regions (Fig 1B), which is comparable to the observed nucleotide composition of genes (39%) in the human genome. On the other hand, it is observed that ~95% and ~84% of pdSNPs and pf-pdSNPs, respectively, reside in non-genic regions, suggesting that these SNPs are enriched in non-genic regions and may play greater roles in affecting gene regulation than directly modifying protein function. Within genes, SNPs are more enriched in the promoter (odds ratio = 1.28, *p-value*<0.001, Fisher’s exact test), coding region (odds ratio = 1.70, *p-value*<0.001, Fisher’s exact test), and 3’UTR (odds ratio = 1.60, *p-value*<0.001, Fisher’s exact test), as compared to intron (odds ratio = 0.69, *p-value*<0.001, Fisher’s exact test) and 5’UTR (odds ratio = 0.43, *p-value*<0.001, Fisher’s exact test) (Fig 1C). Compared to all SNPs, pdSNPs are enriched in 3’UTR (odds ratio = 1.49, *p-value*<0.001, Fisher’s exact test) and intron (odds ratio = 1.20, *p-value*<0.001, Fisher’s exact test). There is higher percentage of pf-pdSNPs residing in the promoter (odds ratio = 5.85, *p-value*<0.001 by Fisher’s exact test), 5’UTR (odds ratio = 5.50, *p-value*<0.001 by Fisher’s exact test), coding regions (odds ratio = 8.25, *p-value*<0.001 by Fisher’s exact test) and 3’UTR (odds ratio = 2.41, *p-value*<0.001 by Fisher’s exact test) compared to pdSNPs (Fig 1C) likely due to current algorithms predicting functionality of SNPs. Meanwhile, the percentage of pdSNPs is quite similar in the various genomic regions except the 3’UTR which has nearly double the proportion of pdSNPs compared to the coding region (Fig 1D, blue bars), which reinforces the notion that differences between different populations may be due to differences in gene regulation rather than protein functions. The proportions of pf-pdSNPs are higher in promoter, 5’UTR, coding and 3’UTR compared to intron.

### Chromosome size does matter in genomic population differentiation

**Fig 2:**
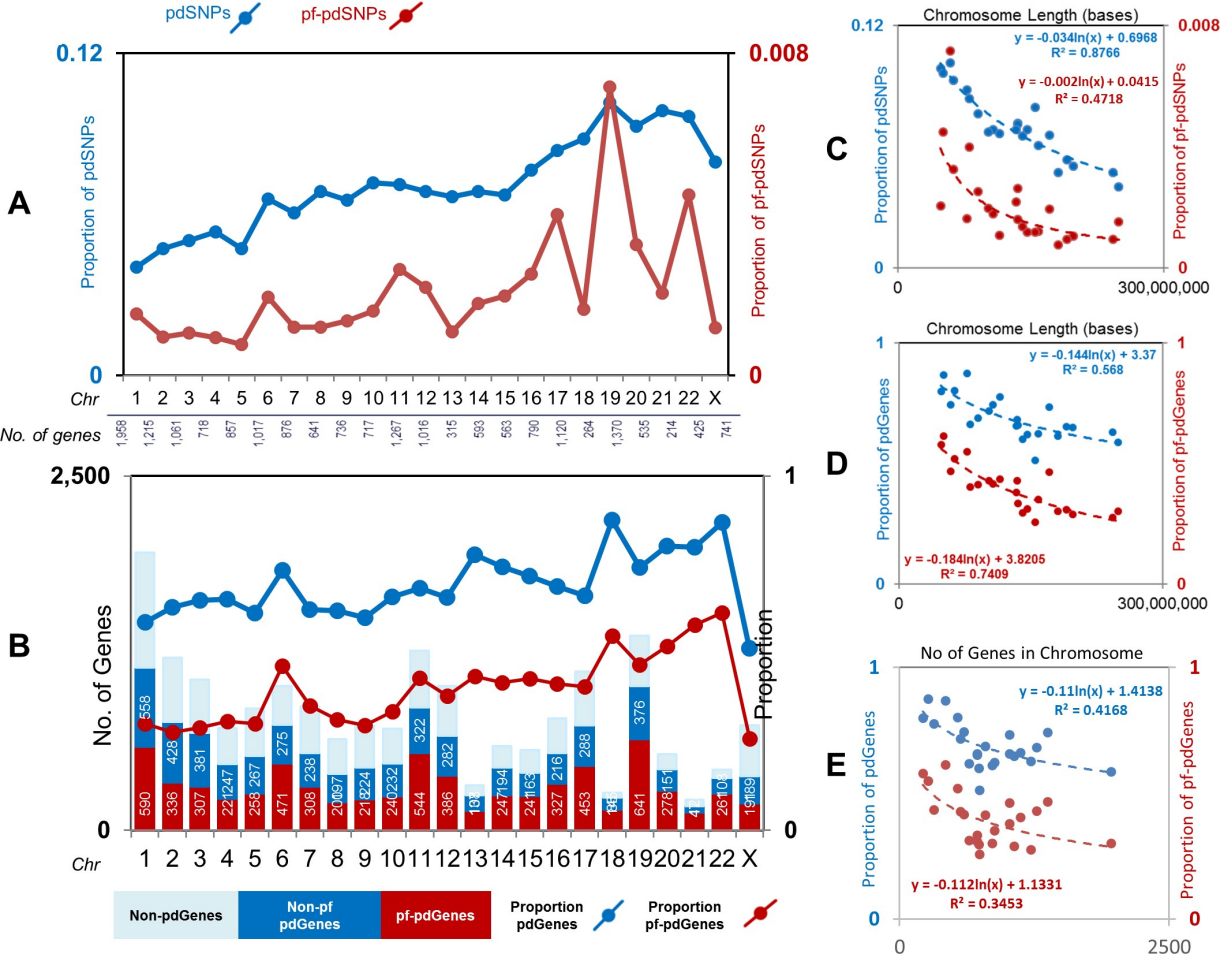
Architecture of pdSNPs and pf-pdSNPs in the human genome. (A) The proportion of pdSNPs (blue line) and pf-pdSNPs (red line) across human chromosomes. (B) The proportion of pdGenes/pf-pdGenes (blue/red lines, right vertical axis), in addition to the number of pdGenes (blue bar) and pf-pdGenes (red bar) out of the total number of all genes (left vertical axis) across the different chromosomes. (C) The correlation between chromosome length and the proportion of pdSNPs and pf-pd SNPs in the respective chromosome. (D) The correlation between chromosome length and the proportion of pdGenes and pf-pdGenes in the respective chromosome. (E) The correlation between the number of genes in the chromosome and the proportion of pdGenes and pf-pdGenes in the respective chromosome.

Chromosomes that have substantial proportion of pdSNPs and pf-pdSNPs may carry clusters of genes under selection pressures that can account for phenotype variation across different populations. To investigate the pattern of population differentiation at the chromosome level, we calculated the proportion of pdSNPs and pf-pdSNPs as well as genes carrying such SNPs, which are referred as the pd-Genes and pf-pdGenes, respectively, across the different chromosomes. Fig 2 shows the proportion of pdSNPs (Fig 2A, blue line) and pf-pdSNPs (Fig 2A, red line), as well as pdGenes (Fig 2B, blue line) and pf-pdGenes (Fig 2B, red line) across different chromosomes, which suggest variable density of such SNPs in the different chromosomes. To control for LD structure across different chromosomes, LD pruning was performed for both pdSNPs and pf-pdSNPs in the CEU population. Similar to our observation using un-pruned SNPs, variable density of pruned pd-/pf-pdSNPs were observed across the different chromosomes, with highest percentage of pruned pd-/pf-pdSNPs being found on chromosome 19 (S1A Fig).

We then determined the relationship between chromosome size and the proportion of the two categories of SNPs. As shown in Fig 2C, there is a reasonable inverse correlation between proportions of pdSNPs (*p-value* = 5.24e-11, R^2^ = 0.8766) and pf-pdSNPs (*p-value* = 2.94e-04, R^2^ = 0.4718) with the length of chromosomes. For the LD-pruned SNP set, the same negative correlation was observed between chromosome length and proportion of pruned pdSNPs (*p-value* = 3.639e-10, R^2^ = 0.8518) and pruned pf-pdSNPs (*p-value* = 2.565e-4, R^2^ = 0.4784) (S1B Fig). Similar inverse correlation was observed between chromosome length and pdGenes (*p-value* = 3.29e-05, R^2^ = 0.568) and pf-pdGenes (*p-value* = 1.37e-07, R^2^ = 0.7409) (Fig 2D, S2B Fig). Moreover, negative correlation was observed between proportion of pdGenes (*p-value* = 8.77e-04, R^2^ = 0.4168) or pf-pdGenes (*p-value* = 3.20e-03, R^2^ = 0.3453) and the number of genes within each chromosome (Fig 2E, S2C Fig). These data suggest that smaller chromosomes tended to have greater proportion of pdSNPS and pf-pdSNPs as well as pdGenes and pf-pdGenes.

### Over-representation of enriched pdGenes and pf-pdGenes across pathways

**Fig 3:**
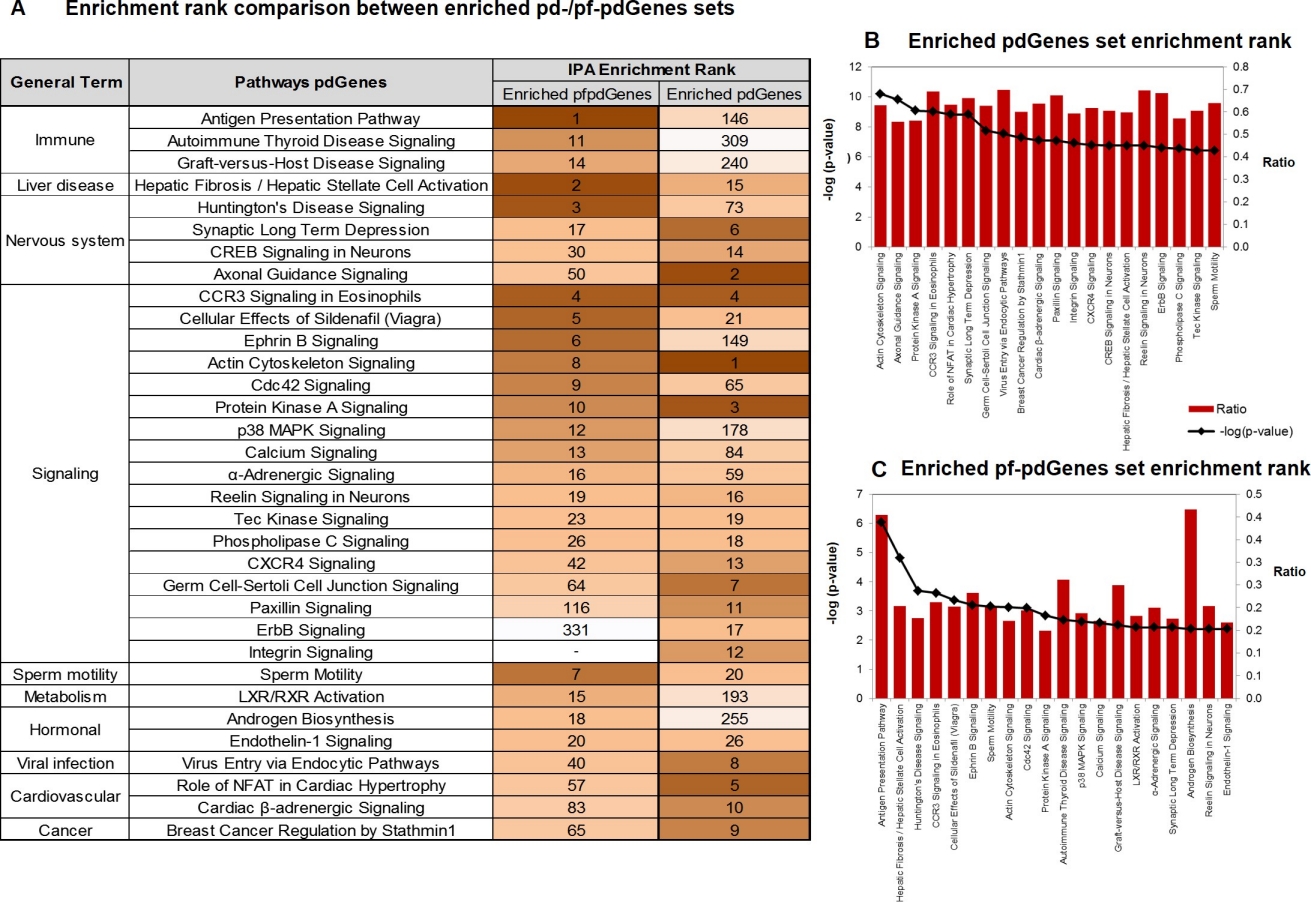
Over-representation of enriched pd-Genes and pf-pdGenes in different canonical pathways. (A) Summary of pathway ranks obtained from Ingenuity^®^ Pathway Analysis (IPA) involving 7,889 enriched pdGenes and 1,906 enriched pf-pdGenes having at least three pdSNPs or pf-pdSNPs. Darkest color (brown) corresponds to the pathway with highest IPA enrichment rank while the lightest color (white) corresponds to the pathway with the lowest IPA enrichment rank. The top 20 pathways that are significantly enriched by pdGenes (B) and pf-pd Genes (C) are also displayed. Ratio denotes the proportion of pdGenes/pf-pdGenes to the total number of genes in the pathways.

To identify pathways, which are significantly affected by pfSNPs and pf-pdSNPs, the Ingenuity^®^ Pathway Analysis (IPA^®^) was employed to interrogate 7,889 enriched pdGenes and 1,906 enriched pf-pdGenes, which contain at least three pdSNPs or pf-pdSNPs, respectively. Interestingly, while Actin Cytoskeleton, Axonal Guidance and Protein Kinase A signaling pathways are enriched with pdGenes (Fig 3A and 3B), Antigen Presentation, Hepatic Fibrosis and Huntington Disease Signalling are over-represented by enriched pf-pdGenes (Fig 3A and 3C).

### Over-representation of enriched pdGenes and pf-pdGenes associated with diseases in different chromosomes

**Fig 4:**
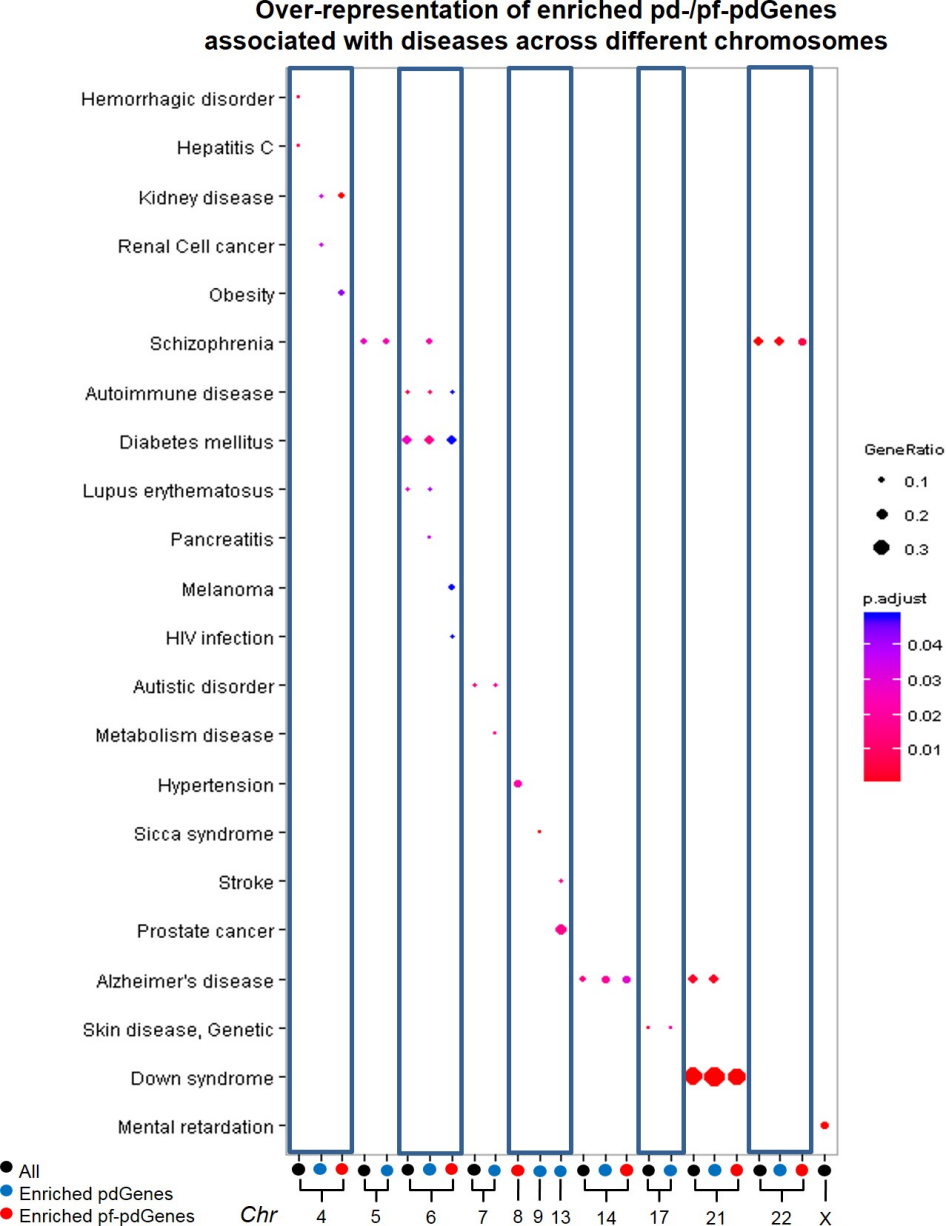
Over-representation of enriched pd- and pf-pdGenes associated with diseases across different chromosomes. The column labelled with black dots below shows over-representation of genes associated with disease term(s) on the specific chromosome(s), while column labelled with red and blue dots included terms over-represented with enriched pdGenes and pf-pdGenes, respectively. The size of the dots inside the column correspond to gene ratio, which represents the proportion of pd/pf-pdGenes to the total number of genes in the pathways, and the color of the dots (blue to red) correspond to the significance of adjusted *p-value* (largest to smallest).

To evaluate the association between phenotypic variation (consequences) and enriched pdGenes and pf-pdGenes in the various chromosomes, we first identified chromosomes that are over-represented in genes associated with specific diseases and then determine if these chromosomes are also over-represented with enriched pdGenes and enriched pf-pdGenes associated with these diseases. As shown in Fig 4, nearly half of the chromosomes (11/23) are over-represented with enriched pd-/pf-pdGenes associated with different diseases. Many of these diseases were reported to have different incidences, severity or manifestation in different ethnic populations (S1 Table).

Concordant with known data [17], genes in chromosome 21 is strongly associated with Down’s Syndrome while genes in chromosome X are associated with Mental Retardation (adjusted *p-value* < 0.05) (Fig 4, S1 Table). Significantly, the data suggests that the Down’s Syndrome genes in chromosome 21 are also significantly enriched with enriched pd-/pf-pdGenes (Adjusted *p-value* < 0.05) while mental retardation genes in chromosome X are not.

Significantly, chromosome 6, which carries the major histocompatibility complex (MHC) loci responsible for immune response, was found to be over-represented with enriched pd-/pf-pdGenes, associated with the most number of different diseases, especially those with immune association including Autoimmune Disease (Adjusted *p-value* < 0.05), Type 1 diabetes (Adjusted *p-value* < 0.05), Lupus Erythematosus (Adjusted *p-value* < 0.05), and HIV infection (Adjusted *p-value* < 0.05). Ethnic differences in these diseases were previously reported [18–22]. Type 1 diabetes was reported to be most common in Northern European, then Southern European and is least common in Asians [19, 20]. In the United States, more African Americans were found to have an increased risk for the development of Systemic Lupus Erythematosus than Caucasians [20, 23]. Ethnic differences in pancreatitis was also reported in the multi-ethnic population of Malaysia where there are significantly more Indians having the disease compared to Chinese [24]. Melanoma, which is often due to ultraviolet (UV) B radiation suppressing the host immune system [25], occurs most frequently in Caucasians compared to other ethnic groups [26]. HIV infection is more likely to affect African Americans compared to Caucasians or Hispanics [22].

Chromosome 8 is significantly over-represented with enriched pf-pdGenes associated with Hypertension (Adjusted *p-value* < 0.05) which is consistent with reports of ethnic variation in Hypertension where Africans develop hypertension at an earlier age, the target organ that is damaged is different from that of the Caucasians and they also respond to different drugs compared to the Caucasians [27]. Chromosome 9 is over-represented with enriched pdGenes associated with Sicca (Sjogren) syndrome (Adjusted *p-value* < 0.05) which is an autoimmune disease presenting with oral and ocular dryness as well as connective tissue disease including rheumatoid arthritis, lupus, scleroderma or polymyositis. Interestingly, Sicca syndrome is more common in non-European (mainly North / sub-Saharan African and Caribbean) (0.016%) compared to Europeans (0.007%) with different disease patterns [28]. Chromosome 13 shows over-representation of enriched pdGenes associated with both Stroke (Adjusted *p-value* < 0.05) and Prostate Cancer (Adjusted *p-value* < 0.05). The etiology of stroke was reported to be different in different populations [29]. Emboli originating from the heart or extracranial large arteries are common in Western populations, whereas small-vessel occlusion or intracranial atherosclerosis is more prevalent in Asians. Prostate cancer was reported to be most prevalent in Africans, less prevalent in European and least prevalent in Asians [30].

## Discussion

Investigation of the population differentiation pattern of the human genome would facilitate a better understanding of the role of population genetic polymorphisms in determining phenotype variation in different populations. Here, we elucidated population genomic differentiation pattern using information derived from SNPs, which are the most abundant variants in the human genome, by computing the *F*_ST_ scores of almost 23 million SNPs in the human genome based on their allele frequency information.

A comparison of the population differentiation pattern of SNPs between the genic and intergenic regions reveal the magnitude of impact of natural selection on the human genome. Significantly, more pdSNPs reside within non-genic regions suggesting these pdSNPs are more likely to affect the regulation of genes rather than affect protein structure or function. One possible explanation is that the less deleterious impact of these non-genic variants results in less functional constraints leading to better ‘survival’ of these non-genic variants. Greater proportion of pf-pdSNPs than pdSNPs are genic. One possibility is that these pf-pdSNPs can serve as potential bridge between genetic population differentiation and variation that can modulate phenotype. These genic pf-pdSNPs can potentially modulate protein function, through affecting 3D structure of proteins [7], protein activity or even post-translational modification. The greater proportion of genic pf-pdSNPs could also be due to the greater availability of algorithms predicting functionality in these regions compared to the other regions.

Relationship between population differentiation and chromosome size revealed an inverse relation between pdSNPs, pf-pdSNPs, pdGenes, pf-pdGenes and chromosome length (Fig 2C and 2D, S1A and S1B Fig). Hence, smaller chromosome carry more SNPs that are potentially functional and population differentiated suggesting that smaller chromosomes may be more receptive to the selective forces that result in population differentiation. This could be related to the chromosome recombination rate, which could be dependent on chromosome size [31]. Nonetheless, it is premature to deduce biological significance from this observation and further in-depth studies have to be performed to help us better understand this.

Knowledge of pathways, molecular functions and diseases that are over-represented with enriched pd-/pfpdGenes can also facilitate the generation of testable hypotheses. Hence, it would be informative to identify pathways, molecular functions and diseases that are over-represented with enriched pd-/pfpdGenes to facilitate our better understanding about diseases that are likely to exhibit population differences in incidence or manifestation as well as about pathways/molecular functions that are likely to be adaptive.

It is significant to note that the antigen presentation pathway shows the most significant over-representation of enriched pf-pdGenes suggesting that they are enriched with variants that are both potentially functional and population differentiated. Similarly, previous studies reported that genetic polymorphisms are associated with differences in immune system and environmental response [32]. Hence, our findings and previous reports are both consistent with the notion that polymorphisms in immune genes would hypothetically be beneficial for host defense mechanism against diverse pathogen variations across different environment [33]. Notably, several immune/infection-related diseases, that were previously reported to exhibit population differences in incidence or manifestation, are significantly over-represented with enriched pd-/pf-pdGenes residing on chromosome 6 (Fig 4, S1 Table**).** It is thus worthwhile to interrogate pf-pdSNPs in pf-pdGenes for their association with these diseases or determine if these pf-pdSNPs were previously reported to be associated with the disease, or in high LD with the associated reported variants identified from previous studies. To gain insights into the role of pf-pdSNPs in these disease/pathways, it is also useful to investigate the molecular function of pd-/pf-pdSNPs in these genes based on the predicted functionality.

In addition to the immune genes, enriched pd-/pf-pdGenes, are significantly over-represented in pathways involving the nervous system (Fig 3). Concordantly, neurological diseases are over-represented by pd-/pf-pdGenes on different chromosomes including chromosome 14 (Alzheimer’s disease), chromosome 21 (Alzheimer’s disease and Down syndrome), chromosome 22 (Schizophrenia),etc. Interestingly, it is also observed that though chromosome 21 and 22 are smaller compared to other chromosomes, they carry significantly high percentage of pf-pdGenes (Fig 2B). Hence, while immune-related pd-/pf-pdSNPs mainly reside on chromosome 6, pd-/pf-pdSNPs enriched in genes associated with neurological diseases are enriched in different chromosomes, especially shorter chromosomes with higher percentage of pd-/pf-pdGenes. These observations suggest that pf-pdSNPs within genes involved in these diseases may play some roles in disease pathology / manifestation. Hence, these pf-pdSNPs should be interrogated for their association with these diseases. It may also be worthwhile to determine if these pf-pdSNPs were previously associated with the disease, or in high LD with variants reported to be associated with the disease. Further characterization of these pf-pdSNPs in these genes will facilitate better design of therapeutic strategies to manage these diseases.

## Conclusions

In summary, interrogating the footprint of population differentiation in the human genome reveals that population differentiated SNPs are more likely to reside in non-genic or regulatory regions of genes suggesting that these SNPs are more likely to modulate gene regulation rather than protein function. Interestingly, greater proportion of pdSNPs/Genes, pf-pdSNPs/Genes are found in smaller chromosomes including chromosome 21 and 22 which show significant over-representation of neurological disease genes enriched with pf-pdSNPs. In addition, genes carrying at least three pf-pdSNPs are enriched in pathways that interact with environment including Antigen Presentation Pathway, auto-immune diseases, viral infection pathways, etc. Notably, several immune/infection-related as well as neurological diseases are enriched with these enriched pd- / pf-pdGenes, suggesting that pd- / pf-pdSNPs may play a role in these diseases warranting further characterization as they may serve as potential predictive markers for these diseases. Future studies could focus on the detailed characterization of pf-pdSNPs of genes in these disease pathways to facilitate the design of better therapeutic strategies.

## Supporting information

S1 Fig Distribution of prune pdSNPs and pf-pdSNPs in the human genome. (A) The proportion of pruned pdSNPs (blue line) and pruned pf-pdSNPs (red line) across human chromosomes. (B) The correlation between chromosome length and the proportion of pruned pdSNPs and pruned pf-pd SNPs in the respective chromosome.
(PDF)

S2 Fig Architecture of pdSNPs and pf-pdSNPs in the human genome. (A) The correlation between chromosome length and the proportion of pdSNPs and pf-pd SNPs in the respective chromosome. (B) The correlation between chromosome length and the proportion of pdGenes and pf-pdGenes in the respective chromosome. (C) The correlation between the number of genes in the chromosome and the proportion of pdGenes and pf-pdGenes in the respective chromosome.
(PDF)

S1 Table Over-representation of enriched pd-/pf-pdGenes associated with different diseases. (PDF)
