# Supplementary material for: Architecture of population-differentiated polymorphisms in the human genome
Source: PLoS One. 2019 Oct 17;14(10):e0224089. doi: 10.1371/journal.pone.0224089 (PMC6797171; doi:10.1371/journal.pone.0224089)
Supplement: S1 Fig — (A) The proportion of pruned pdSNPs (blue line) and pruned pf-pdSNPs (red line) across human chromosomes. (B) The correlation between chromosome length and the proportion of pruned pdSNPs and pruned pf-pd SNPs in the respective chromosome. (PDF) [file pone.0224089.s001.pdf]

S1 Fig

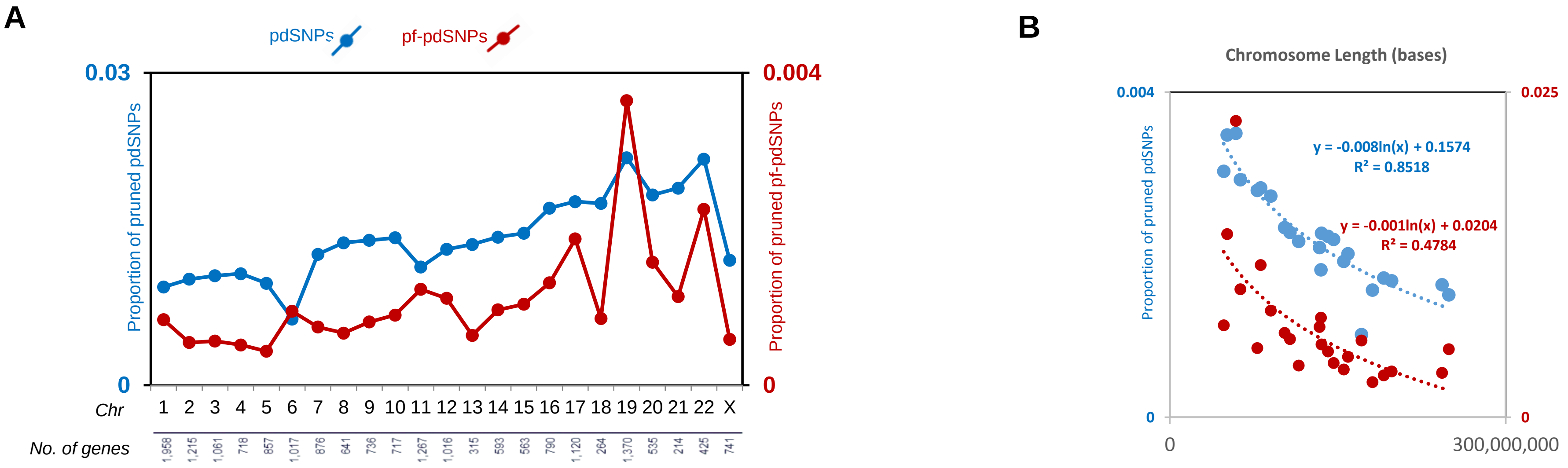

S1 Fig. Distribution of prune pdSNPs and pf-pdSNPs in the human genome. (A) The proportion of pruned pdSNPs (blue line) and pruned pf-pdSNPs (red line) across human chromosomes. (B) The correlation between chromosome length and the proportion of pruned pdSNPs and pruned pf-pd SNPs in the respective chromosome.
