# Supplementary material for: Architecture of population-differentiated polymorphisms in the human genome
Source: PLoS One. 2019 Oct 17;14(10):e0224089. doi: 10.1371/journal.pone.0224089 (PMC6797171; doi:10.1371/journal.pone.0224089)
Supplement: S2 Fig — (A) The correlation between chromosome length and the proportion of pdSNPs and pf-pd SNPs in the respective chromosome. (B) The correlation between chromosome length and the proportion of pdGenes and pf-pdGenes in the respective chromosome. (C) The correlation between the number of genes in the chromosome and the proportion of pdGenes and pf-pdGenes in the respective chromosome. (PDF) [file pone.0224089.s002.pdf]

S2 Fig

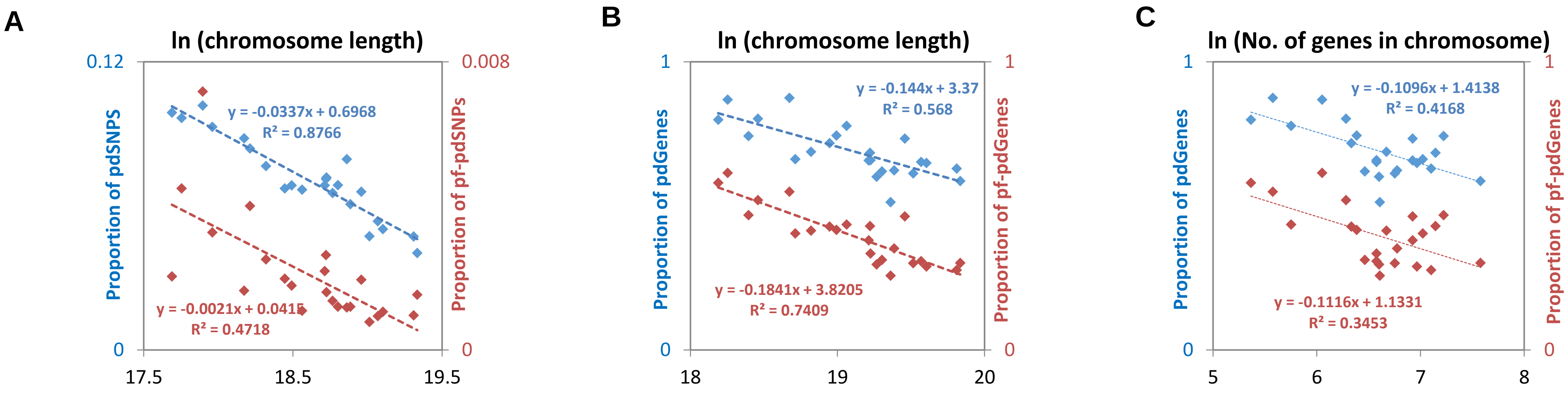

S2 Fig. Architecture of pdSNPs and pf-pdSNPs in the human genome. (A) The correlation between chromosome length and the proportion of pdSNPs and pf-pd SNPs in the respective chromosome. (B) The correlation between chromosome length and the proportion of pdGenes and pf-pdGenes in the respective chromosome. (C) The correlation between the number of genes in the chromosome and the proportion of pdGenes and pf-pdGenes in the respective chromosome.
