# Supplementary material for: Architecture of population-differentiated polymorphisms in the human genome
Source: PLoS One. 2019 Oct 17;14(10):e0224089. doi: 10.1371/journal.pone.0224089 (PMC6797171; doi:10.1371/journal.pone.0224089)
Supplement: S1 Table — (PDF) [file pone.0224089.s003.pdf]

S1 Table. Over-representation of enriched pd-/pf-pdGenes associated with different diseases

Disease	Chromosome over-prepresented by enriched pd-/pf-pdGenes associated with the diseases	Representative Published Reports	Representative References
Kidney Disease	4	End-Stage Kidney Disease (most->Least): African Americans -> Hispanic Americans- >Non-Hispanic Whites	Alves and Lewis (2010). Clinical Nephrology 74:S72-77. 2. Peralta, et al (2011). J Am Soc Nephrol. 22(7):1327-1334
Renal Cell Cancer	4	White more clear cell RCC while blacks more papillary RCC	Olshan , et al (2013) Cancer Medicine 2(5): 744-749
Obesity	4	Black, Mexicans and White women and children, Native Americans, Pacific Island - more affected by obesity while Asian Americans lower prevalence of obesity	Wang and Beydoun (2007) Epidemiologic Reviews 29:6-28
Schizophrenia	5, 6, 22	African Americans are ~3-fold more likely than whites to be diagnosed with schizophrenia	Bresnahan, et al (2007). International Journal of Epidemiology 36:751-758
Autoimmune Diseases	6	Example: Autoimmune hepatitis - In the USA, African-American patients have cirrhosis more commonly, treatment failure more frequently and higher mortality than white American patients. Survival is poorest in Asian-American patients.	Czaia (2013) Expert Rev Gastrenterol Hepatol 7(4): 365-385
Diabetes	6	Type 1 diabetes is most common in Northern European, compared to Southern European, Africans or Asians	Karvonen, et al (2000) Diabetes Care 23: 1516-1526; Cooper and Stroehla (2003) Autoimmunity Reviews 2:119-125
Lupus Erythromatosus	6	Systemic Lupus Erythromatosus (SLE) is more frequent with less favorable outcome in non-white population	Gonzalez, et al (2014) Rheum Dis Clin North Am 40(3):433-454
Pancreatitis	6	Greater percentage of Indians have pancreatitis compared to Malays or Chinese in Perak, Malaysia	Kandasami, et al (2002) Singapore Med Journal 43(6):284-288
Melanoma	6	occurs most frequently in whites than in people of color	Bradford (2009). Dermatol Nurs 21(4): 170-178
HIV Infection	6	African Americans are more likely to be diagnosed with AIDS than Whites or Hispanics	Kraut-Becher, et al (2008) 47:S20-S27
Autistic Disorder	7	Autism prevalence is highest in non-Hispanic white children, lower in Hispanic and African America/black children and highly variable in Asian/Pacific Islander. Higher risk of severe autism phenotypes in children of blacks, South American compared to whites	see Review: Becerra, et al (2014) Pediatrics 134(1): e63
Metabolism Disease	7	Example: Opoid Metabolism - Altered morphine metabolim where Chinese have higher clearance and lower concentrations of morphine	See Review: Smith (2009) Mayo Clinic Proc 84(7): 613-624
Hypertension	8	prevalence of hypertension was highest among non-Hispanic black adults (42.1%), compared with non-Hispanic white (28.0%), Hispanic (26.0%), and non-Hispanic Asian (24.7%) adults	see Review: Rodrique and Ferdinand (2015) Advances in Chronic Kidney Disease 22(2): 145-153
Sicca Syndrome	9	Sicca, also known as Primary Sjogren Syndrome (pSS) is ~2x as common in non-European (mainly N or sub-Saharan African and Caribbean) as Europeans	See Review: Piram, et al (2012) Curr Opin Rheumatol 24:193-200
Stroke	13	Etiology of Stroke Differs in diffeent races - Emboli originating from the heart or extracranial large arteries are common in Western populations, whereas small-vessel occlusion or intracranial atherosclerosis is more prevalent in Asians.	Kim and Kim (2014) Journal of Stroke 16(1):8-17
Prostate Cancer	13	Most prevalent in Africans, less prevalent in European and least prevalent in Asians	Rebbeck and Haas (2014). The Canadian Journal of Urology 21(5): 7496 - 7506
Alzheimer's Disease	14, 21	In the US, blacks and Hispanics are more likely to be afflicted with Alzheimer's Disease compared to Whites	Chin, et al (2011). Alzheimer Dis Assoc Disord 25(3): 18-195
Inflammatory Skin Disease (Genetic)	17	Blacks and Asian children have increased risk of Eczema compared to Hispanic children.	See Review: Kimball (2008). Journal of Investigative Dermatology 13:2-5
Down Syndrome	21	The odds ratio and population attributable risk of Down syndrome due to maternal age of 35 years or older were highest for Mexican Americans, intermediate for African Americans, and lowest for non-Hispanic Whites	Khoshnood, et al (2000) 90(11):1779-1781
